# Pitching into Nicodemus

**Published:** 1854-04

**Authors:** 


### PITCHING INTO NICODEMUS.

A celebrated character of the State of New York, holding a high post in the law, was lately taken ill and confined to his bed for several days. His wife proposed to read for him to which he readily assented.

"My dear, what shall I read?"

"Oh, I don't care much what, anything you please."

"But have you no choice?"

"None in the world, love; please yourself."

"Shall I read a chapter or two out of the Scriptures?"

"Oh, yes, that'll do very well."

"But what part of the Scripture shall I read?"

"Any part you like, love."

"But, you must have some choice, some little preference; we all have that."

"No, I have none in the world; read any part you like best."

"But I would rather please you, dear, and you surely have a preference."

"Well, well, dear; if you will please me, then, *pitch intc Nicodemus.*"

